# Fault Diagnosis Method for Rolling Bearings Based on Grey Relation Degree

**DOI:** 10.3390/e26030222

**Published:** 2024-02-29

**Authors:** Yulin Mao, Jianghui Xin, Liguo Zang, Jing Jiao, Cheng Xue

**Affiliations:** 1 School of Automotive and Rail Transit, Nanjing Institute of Technology, Nanjing 211167, China; y00450220302@njit.edu.cn (Y.M.); xjh2313778@sohu.com (J.X.); y00450220402@njit.edu.cn (J.J.); y00450220226@njit.edu.cn (C.X.); 2 State Key Laboratory of Automotive Simulation and Control, Jilin University, Changchun 130015, China

**Keywords:** rolling bearings, fault diagnosis, subtraction-average-based optimizer, variational mode decomposition, grey relational degree, minimum envelope entropy

## Abstract

Aiming at the difficult problem of extracting fault characteristics and the low accuracy of fault diagnosis throughout the full life cycle of rolling bearings, a fault diagnosis method for rolling bearings based on grey relation degree is proposed in this paper. Firstly, the subtraction-average-based optimizer is used to optimize the parameters of the variational mode decomposition algorithm. Secondly, the vibration signals of bearings are decomposed by using the optimized results, and the feature vector of the intrinsic mode function component corresponding to the minimum envelope entropy is extracted. Finally, the grey proximity and similarity relation degree based on standard distance entropy are weighted to calculate the grey comprehensive relation degree between the feature vector of vibration signals and each standard state. By comparing the results, the diagnosis of different fault states and degrees of rolling bearings is realized. The XJTU-SY dataset was used for experimentation, and the results show that the proposed method achieves a diagnostic accuracy of 95.24% and has better diagnosis performance compared to various algorithms. It provides a reference for the fault diagnosis of rolling bearings throughout the full life cycle.

## 1. Introduction

As a key component of rotating machinery, rolling bearings are widely used in vehicles, aerospace equipment, wind power generation equipment, and so on. They are required to ensure high-precision continuous operation in various complex and harsh environments. However, continuous operation under high load and variable working conditions can easily cause various faults in rolling bearings, leading to equipment shutdown and affecting the production process [1,2,3]. Therefore, how to effectively extract fault features from the full life cycle data of rolling bearings and accurately diagnose different fault states and degrees is of great practical significance for ensuring the safe and stable operation of mechanical equipment.

At present, the commonly used methods of fault feature extraction include wavelet transform (WT), empirical mode decomposition (EMD), and Hilbert–Huang transform (HHT). However, these traditional methods all have certain shortcomings. WT is not suitable for processing non-stationary signals because it is prone to losing high-frequency information in the signals during feature extraction. Therefore, most studies combine WT with other methods. To identify various bearing defects of electric vehicle engines, Choudhary et al. [4] used the wavelet synchrosqueezing transform method to decompose vibration signals and convert them into time–frequency representations. Subsequently, they used a convolutional neural network for fault diagnosis. Liang et al. [5] combined WT with semi-supervised generative adversarial nets to solve the problem of the high cost of marking all samples manually in traditional methods.

Compared with WT, EMD and HHT can effectively analyze non-stationary signals, but there are serious problems with modal aliasing and endpoint effects [6,7]. Therefore, the variational mode decomposition (VMD) method has been proposed, which transforms the signal decomposition into a problem of solving the optimal solution of a constrained model. After extracting the feature vectors of rolling bearings using VMD, Habbouche et al. [8] inputted them into a convolution neural network to realize multi-scale feature extraction triggered by pooling layers and complete the fault diagnosis. Heydari et al. [9] combined VMD with a multi-verse optimization algorithm to process the temperature signals of bearings and lubricating oil in a wind turbine gearbox. Then, they detected abnormal situations based on Mahalanobis distances and the WT method.

Although the VMD method can effectively avoid endpoint effects and suppress modal aliasing, the parameter secondary penalty factor and mode number need to be preset artificially. Due to the sensitivity of the results to parameters, the accuracy of VMD is greatly limited. For this reason, Wang et al. [10] used the genetic mutation particle swarm optimization algorithm to optimize VMD and created a fitness function with cyclic information entropy to find the best parameters. Lv et al. [11] took the minimum average envelope entropy as the objective function and used the grey wolf optimization (GWO) algorithm to adaptively search for the optimal parameters of VMD, avoiding the under-decomposition or over-decomposition problems caused by improper parameter settings.

After extracting fault features, a pattern recognition technology is needed to achieve an automated diagnosis of the fault states of rolling bearings. Currently, the most widely used methods are Back Propagation (BP) and Support Vector Machine (SVM) [12,13]. However, the training of BP requires many samples containing fault features, making it challenging to implement in practical applications. Jia et al. [14] selected 1200–1600 sets of samples from the IMS bearing dataset as the initial faults for bearing degradation state and inputted them into the BP prediction model for training. Cao et al. [15] used the publicly available dataset of rolling bearings provided by Case Western Reserve University for method validation. A total of 160 samples were selected under each bearing load, and an experimental dataset was constructed with 3000 data points per sample length. VMD was uniformly used for decomposition and then input into the BP neural network model for diagnosis, which required a relatively long diagnostic time. Although the SVM method is suitable for training with small samples, the accuracy depends on the selection of optimal parameters. To ensure diagnostic accuracy, it is necessary to incorporate optimization algorithms and/or design complex multi-class structures. Liu et al. [16] improved the SVM method by combining the gravitational search algorithm to optimize the radii of the penalty function and the kernel function. Considering that the arithmetic optimization algorithm has the advantages of fast convergence speed, high precision, and strong randomness [17], Chen et al. [18] applied it to optimize their SVM model, solving the problems of identifying single and composite faults.

As an important basis for the application of artificial intelligence technology, hardware equipment can effectively improve its computing power and promote the continuous optimization of various diagnostic algorithms. Limited by the information processing ability of hardware equipment, Zhu et al. [19] improved the strip attention mechanism so that the network model focused on the important features of training resources and ignored the secondary information to fully extract the time series characteristics of rolling bearing data and realize the identification of different fault types. Although the fault diagnosis performance of this method is better than that of SVM and BP models, it is more dependent on the computing power of hardware equipment, and the calculation is more complicated.

Given the aforementioned issues, a fault diagnosis method based on small sample training and a simpler structure is urgently needed. As a new field of control theory, grey system theory focuses on the study of uncertain systems with small samples and poor information, which is characterized by having some known information and some unknown information [20]. As an important concept in grey system theory, grey relation analysis evaluates the relationships between different sequences based on the geometric shape of sequence curves. The closer the curve is, the higher the relation degree, and vice versa [21]. Due to its fast computation and low redundancy, it has been used in the field of fault diagnosis [22]. Lian et al. [23] used the deep denoising autoencoder method to extract features from bearing vibration signals. Then, the early failure time of bearings was determined by calculating the grey relation degree between the feature vector of vibration signals and normal state as an indicator of bearing performance degradation and utilizing a 3σ threshold. However, the diagnosis of the later fault degree of the bearings was not realized. Aiming at the problem of fault diagnosis in transformers, Peng et al. [24] combined grey relation analysis with a BP network, fuzzy genetic algorithm, and artificial neural network.

Inspired by the aforementioned research findings, this paper proposes a fault diagnosis method for rolling bearings based on grey relation degree to effectively extract various fault features of vibration signals and achieve the recognition and diagnosis of different fault states and degrees. The main contributions are summarized as follows:

(1) Considering the problem of under-decomposition or over-decomposition caused by pre-setting VMD parameters artificially, this paper takes the minimum envelope entropy as the objective function and optimizes VMD through the subtraction-average-based optimizer (SABO) to suppress modal aliasing and endpoint effects.
(2) Aiming at the problem that the traditional grey relation model cannot accurately reflect the similarity and proximity between two curves, this paper uses the weighted grey proximity and similarity relation model based on standard distance entropy to calculate the grey comprehensive relation degree between the feature vector of vibration signals and each standard state. By comparing the calculation results, the diagnosis of different fault states and degrees of rolling bearings is realized.
(3) To verify the effectiveness and accuracy of the proposed method, the XJTU-SY rolling element bearing accelerated life test datasets were used for testing. By comparing various methods of diagnosis, it can be concluded that the proposed method has the highest diagnostic accuracy, has the most effective recognition, and solves the problems of the existing bearing fault diagnosis methods requiring a large number of training samples and the establishment of complex structural network models.

The structure of the remaining parts of this paper is as follows: Section 2 provides an overview of the basic theory of the VMD algorithm and how to optimize VMD using SABO to decompose the fault signals of rolling bearings. Section 3 elaborates on the method of extracting feature vectors and how to use the grey relation degree model based on standard distance entropy to achieve bearing fault diagnosis. Section 4 applies the publicly available XJTU-SY datasets for experimental verification and compares the accuracy of different methods. Section 5 presents some conclusions and directions for future work.

## 2. Decomposition of Fault State Signals

### 2.1. VMD Algorithm

In complex working environments, the vibration signals of rolling bearings are inevitably affected by background noise and other coupling components [25], resulting in the obtained signals being non-linear, non-stationary, and non-Gaussian and having characteristics such as a low signal-to-noise ratio. To better recognize the states of rolling bearings and improve the accuracy of fault diagnosis, it is necessary to denoise the vibration signals before extracting the feature vectors [26]. The VMD method was proposed by Konstantin Dragomiretskiy and other scholars in 2014 [27], and it is widely used in signal processing in various fields. It has significant advantages in analyzing non-linearity and non-stationary signals and effectively suppresses the problems of endpoint effects and modal aliasing [28]. It mainly includes two parts—the construction and solution of constrained variational problems—and the specific implementation process is summarized as follows:

(1) Construction of constrained variational problems

(1) Assuming there is a signal f(t) to be decomposed, consisting of multiple modal component signals $u_{k}(t)$:

(1) $$f(t)=\sum_{k=1}^{K}u_{k}(t)=\sum_{k=1}^{K}A_{k}(t)\cos[\varphi_{k}(t)]$$

where *K* is the total number of modal components, *k* is the index of the modal component signal, $A_{k}(t)$ is the amplitude of $u_{k}(t)$, and $\varphi_{k}(t)$ is the phase angle of $u_{k}(t)$.

(2) Demodulate each modal component signal through Hilbert transform to obtain the unilateral spectrum $h_{k}(t)$:

(2) $$h_{k}(t)=[\delta (t)+\frac{j}{\pi t}]*u_{k}(t)$$

where δ(t) is the unit impulse function, *t* is the time variable of the vibration signal, *j* is the imaginary unit, and ∗ is the convolution operation.

(3) Modulate the spectrum of each demodulated signal onto the corresponding baseband and construct the response function of the demodulator $h_{k}^{\prime }(t)$:

(3) $$h_{k}^{\prime }(t)=\left[\left(\delta (t)+\frac{j}{\pi t}\right)*u_{k}(t)\right]e^{-j\omega_{k}t}$$

where $e^{-j\omega_{k}t}$ is the estimated center frequency, $\omega_{k}$ is the frequency parameter of the *k*-th modal component signal, and *e* is a natural constant.

(4) Take the square norm of the demodulation signal gradient mentioned above and estimate the bandwidth corresponding to each modal component to obtain the constrained variational problem:

(4) $$M^{\prime }=\underset{\left\{u_{k}\right\},\left\{\omega_{k}\right\}}{\min}\sum_{k}{\left\|\frac{\partial }{\partial t}\left\{\left[\delta (t)+\frac{j}{\pi t}\right]*u_{k}(t)\right\}e^{-j\omega_{k}t}\right\|}_{2}^{2}$$

where $M^{\prime }$ is the minimum value of the sum of squared norms of gradients corresponding to all modal components, $\left\{u_{k}\right\}$ is the set of all modal component signals, and $\left\{\omega_{k}\right\}$ is the set of frequency parameters of all modal component signals.

(2) Solution of constrained variational problems

(1) To solve constrained variational problems, it is necessary to introduce a secondary penalty factor and a Lagrangian penalty operator. If there is Gaussian white noise in the signal, the former can ensure the reconstruction accuracy of the signal, while the latter can strictly constrain the variational model. The constructed Lagrangian augmented function is as follows:

(5) $$L\left[\left\{u_{k}\right\},\left\{\omega_{k}\right\},\lambda \right]=\alpha \sum_{k}{\left\|\frac{\partial }{\partial t}\left\{\left[\delta (t)+\frac{j}{\pi t}\right]*u_{k}(t)\right\}e^{-j\omega_{k}t}\right\|}_{2}^{2}+{\left\|f\left(t\right)-\sum_{k}u_{k}(t)\right\|}_{2}^{2}+\langle \lambda \left(t\right),f\left(t\right)-\sum_{k}u_{k}(t)\rangle$$

where *L*[·] is the augmented Lagrangian function transformation, λ is the Lagrangian multiplication operator, α is the secondary penalty factor, <·> represents the inner product operation, and λ(t) is the Lagrangian multiplication operator at time *t*.

(2) For Equation (5) above, the optimal solution to the variational problem can be obtained by constantly updating the values of $u_{k}^{n+1}$, $\omega_{k}^{n+1}$, and $\lambda^{n+1}$:

(6) $$u_{k}^{n+1}=\underset{u_{k}\in X}{\arg\min}L\left(\left\{u_{k}\right\},\left\{\omega_{k}\right\},\lambda \right)=\underset{u_{k}\in X}{\arg\min}(\alpha \sum_{k}{\left\|\frac{\partial }{\partial t}\left\{\left[\delta (t)+\frac{j}{\pi t}\right]*u_{k}(t)\right\}e^{-j\omega_{k}t}\right\|}_{2}^{2}+{\left\|f\left(t\right)-\sum_{k}u_{k}(t)+\frac{\lambda \left(t\right)}{2}\right\|}_{2}^{2})$$

where $u_{k}^{n+1}$ is the updated result of the *k*-th modal component signal in the *n* + 1 iteration, and *X* is the entire signal space.

(3) Equation (6) is transformed from the time domain to the frequency domain with the use of Fourier transform, and the corresponding extremum is solved to obtain the modal component ${\hat{u}}_{k}$ and the corresponding center frequency $\omega_{k}$:

(7) $${\hat{u}}_{k}^{n+1}\left(\omega \right)=\frac{\hat{f}\left(\omega \right)-\sum_{i\neq k}{\hat{u}}_{i}\left(\omega \right)+\frac{\hat{\lambda }\left(\omega \right)}{2}}{1+2\alpha {\left(\omega -\omega_{k}\right)}^{2}}$$

(8) $$\omega_{k}^{n+1}=\frac{\int_{0}^{\infty }\omega {\left|{\hat{u}}_{k}\left(\omega \right)\right|}^{2}d\omega }{\int_{0}^{\infty }{\left|{\hat{u}}_{k}\left(\omega \right)\right|}^{2}d\omega }$$

(4) Update the Lagrangian multiplication operator λ:

(9) $${\hat{\lambda }}^{n+1}(\omega )={\hat{\lambda }}^{n}(\omega )+\tau \left[\hat{f}(\omega )-\sum_{k}{\hat{u}}_{k}^{n+1}(\omega )\right]$$

where τ is the noise tolerance parameter.

(5) Set the convergence threshold ε>0 to determine whether the convergence condition shown in Equation (10) below is satisfied. If it is, stop the decomposition. If it is not satisfied, let n=n+1 and return to step (3) to continue the decomposition:

(10) $$\sum_{k}\frac{\left\|{\hat{u}}_{k}^{n+1}(t)-{\hat{u}}_{k}^{n}(t)\right\|}{{\left\|{\hat{u}}_{k}^{n}(t)\right\|}_{2}^{2}}<\varepsilon$$

(6) Perform inverse Fourier transform on the iterative results ${\hat{u}}_{k}^{n}$ to obtain the number of *k* intrinsic mode function (IMF) components with a certain bandwidth and fluctuating around the center frequency.

### 2.2. SABO Algorithm Optimizes VMD

When solving constrained variational problems, the mode number *k* and secondary penalty factor α of the VMD decomposition need to be set in advance. If *k* is less than the number of useful components in the signal to be decomposed, it will lead to insufficient decomposition results and the phenomenon of modal aliasing. If *k* is greater than the number of useful components in the signal to be decomposed, false components will be generated. The secondary penalty factor α determines the bandwidth of each IMF component. A smaller α will result in a larger bandwidth, leading to some components containing signals from other components. On the contrary, a larger α will result in a smaller bandwidth of each IMF component, leading to the loss of certain signals in the decomposed signal. In order to find the optimal *k* and α, this paper proposes using the SABO algorithm to optimize VMD.

The SABO algorithm is a new intelligent optimization algorithm proposed by Trojovský Pavel and Dehghani Mohammad in 2023 [29]. The process of parameter optimization is as follows:

(1) Calculate the value of the target decision variable for the target search agent in the search space:

(11) $$x_{i,d}=lb_{d}+r_{i,d}\cdot (ub_{d}-lb_{d})\;i=1,\cdots ,N,\;d=1,\cdots ,m$$

where *N* is the number of search agents, *m* is the number of decision variables, $x_{i,d}$ is the value of the *d*-th decision variable of the *i*-th search agent in the search space, $r_{i,d}$ is the random number of the *d*-th decision variable of the *i*-th search agent in the search space (its value is between [0,1]), and $lb_{d}$ and $ub_{d}$ are the lower and upper optimization boundaries of the *d*-th decision variable, respectively.

(2) Calculate the new proposal position for each search agent in SABO:

(12) $$X_{i}-vX_{j}=sign(F(X_{i})-F(X_{j}))(X_{i}-\vec{v}\cdot X_{j})$$

(13) $$X_{i}^{new}=X_{i}+\vec{r_{i}}*\frac{1}{N}\sum_{j=1}^{N}\left(X_{i}-vX_{j}\right)\;i=1,2,\cdots ,N$$

where v is a special operator symbol, and $\vec{v}$ is a vector with dimension *m*, where the components are random numbers generated from the set {1,2}. $X_{i}^{new}$ is the new proposed position of the *i*-th search agent $X_{i}$, and $\vec{r_{i}}$ is a vector with dimension *m* of the *i*-th search agent, where the components have a normal distribution, and their values come from the interval [0,1]. $X_{j}$ is the *j*-th search agent.

(3) Construct constraints for updating the proposed position. During the iteration process, if the new proposed position results in a higher value of the objective function, it can serve as the new position for the corresponding agent. Otherwise, it remains unchanged. The calculation equation is as follows:

(14) $$X_{i}=\left\{ \begin{array}{l} X_{i}^{new},\;F_{i}^{new}<F_{i}\\ X_{i},\;\;else \end{array}\right.$$

where $F_{i}$ and $F_{i}^{new}$ are the objective function values of the search agent $X_{i}$ and $X_{i}^{new}$, respectively.

To verify the optimization performance of the SABO algorithm, the CEC2005 optimization function test set was selected for testing. The single-peak test functions F1 and F2 were used to test the optimization accuracy and speed of the algorithm, and the multi-peak test functions F8 and F9 were used to test the global optimization ability of the algorithm and whether it can jump out of the optimal solution. The expressions and search spaces of the test functions are shown in Table 1.

**Figure 1:**
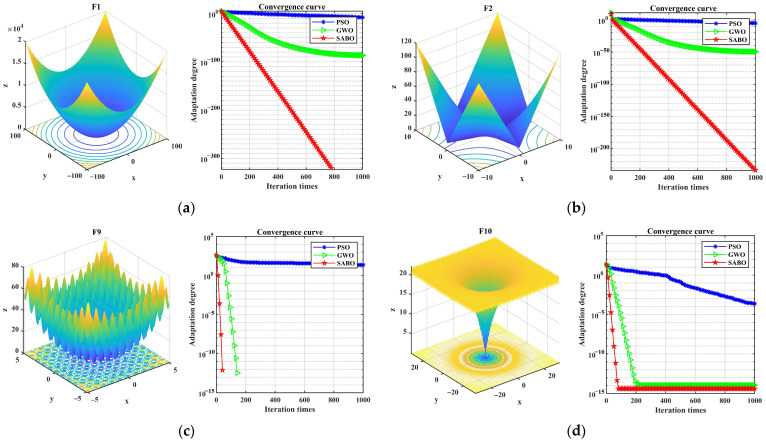
Convergence curves of SABO, PSO, and GWO algorithms under different test functions. (**a**) Image and convergence curve of F1; (**b**) image and convergence curve of F2; (**c**) image and convergence curve of F9; (**d**) image and convergence curve of F10.

The SABO, particle swarm optimization (PSO), and GWO algorithms are used for iterative calculations, respectively. The iteration numbers were set to 1000, and the population sizes were uniformly set to 100. The comparison results are shown in Figure 1. Figure 1a–d show three-dimensional images of functions F1, F2, F9, and F10, as well as the convergence curves of the testing algorithm. It can be seen from Figure 1 that the SABO algorithm reaches the optimal value first, indicating that its optimization accuracy, speed, and global optimization ability are superior to the PSO and GWO algorithms.

**Figure 2:**
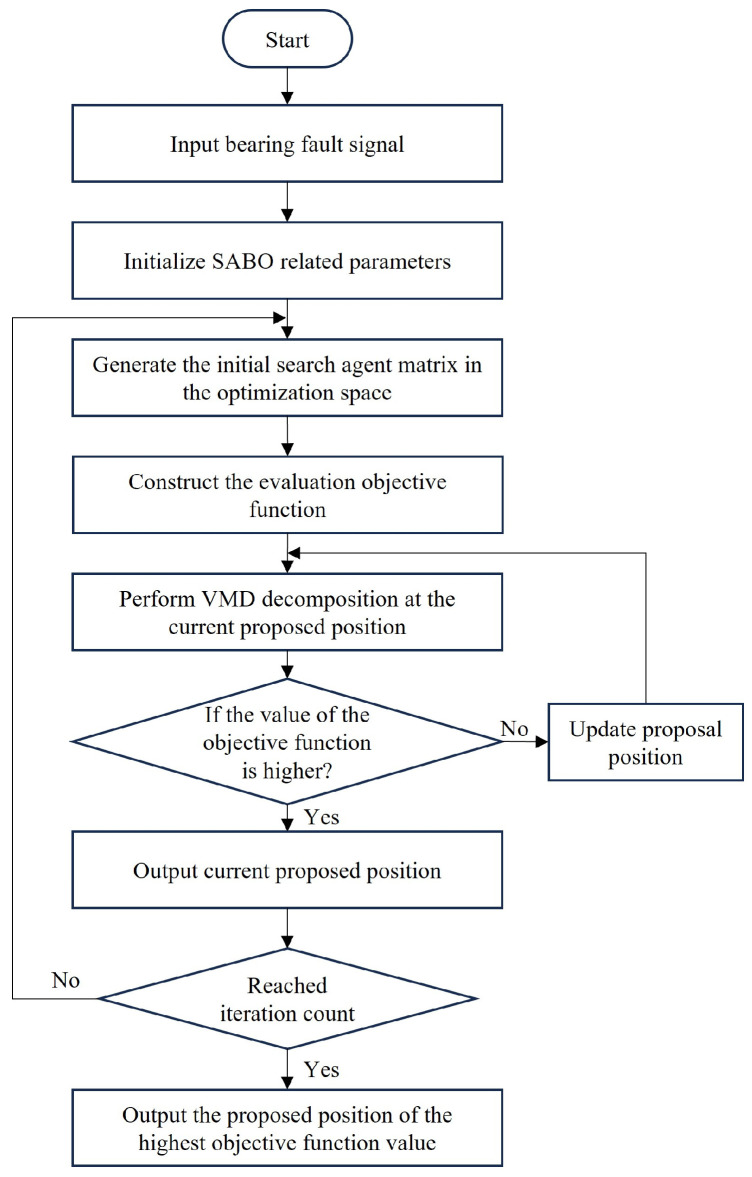
Flow chart of using the SABO algorithm to optimize VMD.

Given the advantages of the SABO algorithm, the authors of this paper used SABO to optimize VMD to find the optimal mode number *k* and secondary penalty factor α with minimum envelope entropy as the objective function. The main implementation steps are as follows: Firstly, the bearing signals are input, the ranges of *k* and α are set, and the relevant parameters of the SABO algorithm are initialized, namely, *D* (the variable number to be optimized), *N* (population number), and *T* (maximum iteration number). Secondly, the initial search agent matrix is randomly generated in the optimization space. Then, the evaluation objective function is constructed, which is the minimum envelope entropy. VMD decomposition is performed at the current proposed position (under current *k* and α) of the search agent to calculate the objective function value. According to Equation (13), the proposed position of the search agent is updated, and whether the new proposed position can make the objective function value higher is judged according to Equation (14). Save the position corresponding to the highest objective function value after an iteration. Finally, the iteration is repeated, and the optimal optimization results after the iteration are output, that is, the mode number *k* and the secondary penalty factor α of VMD decomposition. The specific process is shown in Figure 2.

## 3. Reconstruction and Diagnosis of Fault Signals

### 3.1. Feature Vector Extraction

The extraction of feature vectors is one of the important steps in bearing fault state recognition. Extracting feature parameters that are sensitive to changes in the health status of the bearing itself is the key to the recognition and diagnosis of rolling bearings [30]. By calculating the energy values of each modal component, the energy distribution of each natural vibration mode can be obtained. Different types of bearing faults often lead to changes in the energy values of specific modes. Therefore, fault features can be extracted by comparing the energy values under different fault conditions to help with fault diagnosis.

Otherwise, the time-domain characteristics of signals can reflect the overall state of the bearing and are widely used for fault diagnosis and trend prediction [31,32]. The time-domain characteristic parameters are divided into dimensional and non-dimensional parameters. The mean, variance, peak, root mean square value, and peak factor, as dimensional parameters, will change over time and can, respectively, reflect the offset of the signal, the degree of dispersion, the amplitude of vibration, the effective amplitude, and the relationship between the peak and the effective amplitude. Through the analysis of eigenvalue data, the fault condition of the bearing can be effectively detected. As non-dimensional parameters, kurtosis, pulse factor, waveform factor, and margin factor can reflect the peak concentration, pulse properties, waveform shape, and the margin between peak and mean of the signal, respectively, effectively representing the real-time condition of the bearing, making up for the problem that the dimensional parameters change due to the change in working conditions, and characterizing the working state of the bearing as a whole.

In order to provide a more comprehensive description and analysis of the overall state and fault characteristics of bearings, this paper takes the IMF component corresponding to the minimum envelope entropy as the optimal IMF component after using the SABO algorithm to obtain the optimal mode number *k* and the secondary penalty factor α for VMD decomposition. For the best IMF component, the mean, variance, peak value, kurtosis, root mean square, peak factor, pulse factor, waveform factor, margin factor, and energy value are calculated in turn. The feature vector of each sample datum is constructed with these ten indicators. The equation of each feature parameter is shown in Table 2.

### 3.2. Grey Relation Degree Model Based on Standard Distance Entropy

Grey relation degree is an important method for the judgment and analysis of grey system theory. Rolling bearing is a dynamic process during service, and there is an inevitable connection between the vibration signals in the early and late stages [33,34]. Therefore, calculating the grey relation degree between the feature vectors of test samples at different periods and standard states can serve as a basis for judging the status and degree of bearing faults.

The traditional grey relation model is as follows: Let the origination zeroing grey generation of sequence $X_{i}=\left\{x_{i}(1),x_{i}(2),\cdots ,x_{i}(n)\right\}$ be $X_{i}^{0}=\left\{x_{i}^{0}(1),x_{i}^{0}(2),\cdots ,x_{i}^{0}(n)\right\}$, where $x_{i}^{0}(k)=x_{i}(k)-x_{i}(1)$. Let the origination zeroing grey generation of sequence $X_{j}=\left\{x_{j}(1),x_{j}(2),\cdots ,x_{j}(n)\right\}$ be $X_{j}^{0}=\left\{x_{j}^{0}(1),x_{j}^{0}(2),\cdots ,x_{j}^{0}(n)\right\}$, where $x_{j}^{0}(k)=x_{j}(k)-x_{j}(1)$ and k=1,2,⋯,n. The grey similarity relation degree based on the similarity perspective is $\varepsilon_{ij}=\frac{1}{1+\left|s_{i}-s_{j}\right|}$, where $s_{i}-s_{j}=\int_{1}^{n}(X_{i}^{0}-X_{j}^{0})dt$. The grey proximity relation degree based on the proximity perspective is $\rho_{ij}=\frac{1}{1+\left|S_{i}-S_{j}\right|}$, where $S_{i}-S_{j}=\int_{1}^{n}(X_{i}-X_{j})dt$.

The more similar the sequence $X_{i}$ and $X_{j}$ are in geometry, the larger the $\varepsilon_{ij}$ is, and vice versa. The closer the sequence $X_{i}$ and $X_{j}$ are in space, the larger the $\rho_{ij}$ is, and vice versa. The traditional grey relation model may have the problem that the grey similarity relation degree of two non-parallel sequences may be 1 and the grey proximity relation degree of two different sequences may be 1, which means it cannot accurately reflect the similarity and proximity between the two curves, resulting in significant errors. Therefore, this paper introduces a grey relation degree model based on standard distance entropy.

Assuming that there are two constant positive sequences, namely $X_{i}=\left\{x_{i}(1),x_{i}(2),\cdots ,x_{i}(n)\right\}$ and $X_{j}=\left\{x_{j}(1),x_{j}(2),\cdots ,x_{j}(n)\right\}$, the grey proximity relation degree based on standard distance entropy is as follows:

(15) $$H_{ij}=\frac{1}{n\ln2}\overset{n}{\underset{t=1}{\Sigma }}[-(\frac{x_{i}\left(t\right)}{x_{i}\left(t\right)+x_{j}\left(t\right)}\ln\frac{x_{i}\left(t\right)}{x_{i}\left(t\right)+x_{j}\left(t\right)}+\frac{x_{j}\left(t\right)}{x_{i}\left(t\right)+x_{j}\left(t\right)}\ln\frac{x_{j}\left(t\right)}{x_{i}\left(t\right)+x_{j}\left(t\right)})]$$

where *n* is the number of elements in the sequence $X_{i}$ and $X_{j}$.

Assuming that there are two sequences, namely $X_{i}=\left\{x_{i}(1),x_{i}(2),\cdots ,x_{i}(n)\right\}$ and $X_{j}=\left\{x_{j}(1),x_{j}(2),\cdots ,x_{j}(n)\right\}$, where $x_{i}(t)$ and $x_{j}(t)$ satisfy $x_{i}\left(t+1\right)>x_{i}\left(t\right)$ and $x_{j}\left(t+1\right)>x_{j}\left(t\right)$, the grey similarity relation degree based on standard distance entropy is as follows:

(16) $$\begin{array}{l} H_{ij}^{\prime }=\frac{1}{\left(n-1\right)\ln2}\sum_{t=1}^{n-1}[-(\frac{x_{i}\left(t+1\right)-x_{i}\left(t\right)}{\left(x_{i}\left(t+1\right)-x_{i}\left(t\right)\right)+\left(x_{j}\left(t+1\right)-x_{j}\left(t\right)\right)}*\ln\frac{x_{i}\left(t+1\right)-x_{i}\left(t\right)}{\left(x_{i}\left(t+1\right)-x_{i}\left(t\right)\right)+\left(x_{j}\left(t+1\right)-x_{j}\left(t\right)\right)}\\ +\frac{x_{j}\left(t+1\right)-x_{j}\left(t\right)}{\left(x_{i}\left(t+1\right)-x_{i}\left(t\right)\right)+\left(x_{j}\left(t+1\right)-x_{j}\left(t\right)\right)}*\ln\frac{x_{j}\left(t+1\right)-x_{j}\left(t\right)}{\left(x_{i}\left(t+1\right)-x_{i}\left(t\right)\right)+\left(x_{j}\left(t+1\right)-x_{j}\left(t\right)\right)})] \end{array}$$

**Figure 3:**
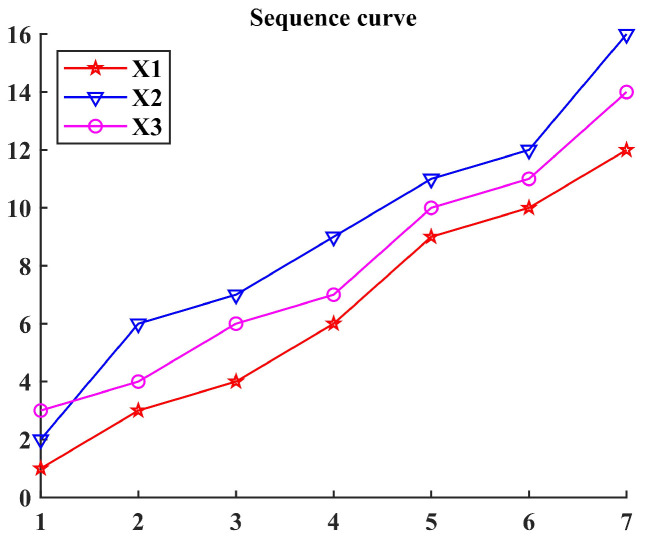
The sequence curves of an example.

An example is given below to illustrate: Consider the sequences $X_{1}=\left\{1,3,4,6,9,10,12\right\}$, $X_{2}=\left\{2,6,7,9,11,12,16\right\}$, $X_{3}=\left\{3,4,6,7,10,11,14\right\}$, as shown in Figure 3. These three sequences are substituted into the grey relation model based on standard distance entropy for calculation. Then, the results—$H_{12}=0.9607$, $H_{13}=0.9650$, $H_{12}^{\prime }=0.9679$, and $H_{13}^{\prime }=0.9543$—can be obtained. Due to $H_{12}<H_{13}$, sequence $X_{3}$ is closer to sequence $X_{1}$ in spatial distance than sequence $X_{2}$; due to $H_{12}^{\prime }>H_{13}^{\prime }$, sequence $X_{2}$ is more similar to sequence $X_{1}$ in geometric shape than sequence $X_{3}$.

### 3.3. Bearing Fault Diagnosis Based on Grey Comprehensive Relation Degree

In order to reflect the spatial distance and shape characteristics between curves simultaneously, it not only reflects the changes in feature vectors of different states but also fully reflects the changes between different feature values of feature vectors. In this paper, the grey proximity and similarity relation degrees based on standard distance entropy are calculated, respectively, and the weighted average is taken as the grey comprehensive relation degree for each state. The closer the value of the grey comprehensive relation degree is to one, the closer the data is to a specific fault state so as to determine the fault situation of the samples and achieve a diagnosis of different fault states and degrees of rolling bearings.

**Figure 4:**
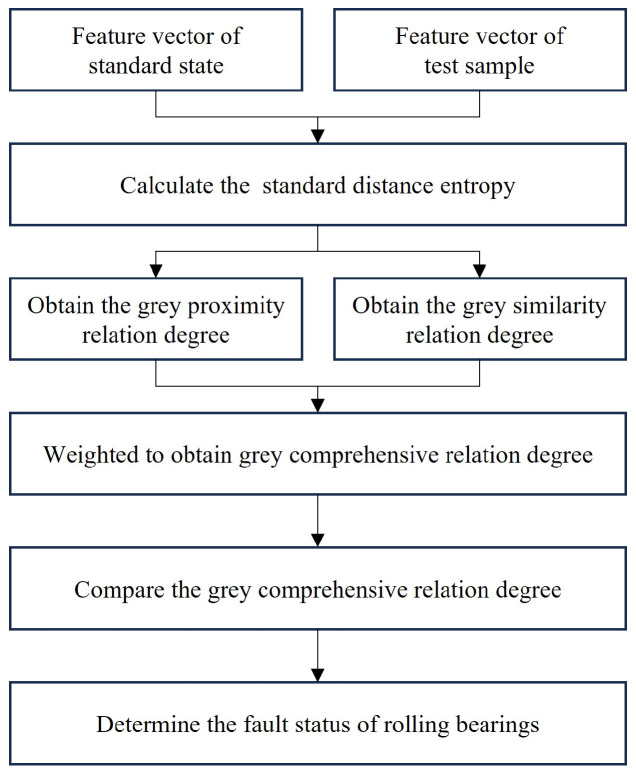
Flow chart of bearing fault diagnosis based on grey comprehensive relation degree.

Firstly, it is necessary to extract the feature vectors of each standard state, test all samples, and calculate the grey proximity relation degree *H_ab_* and grey similarity relation degree *H*′*_a_*_′*b*′_ based on the standard distance entropy between the feature vector of each test sample and standard state. Secondly, the *H_ab_* and *H*′*_a_*_′*b*′_ calculated for each test sample are weighted to obtain the grey comprehensive relation degree. Finally, the grey comprehensive relation degree between each test sample and standard state is compared to obtain the diagnosis results of the fault states. The specific process is shown in Figure 4.

## 4. Experimental Verification

### 4.1. Fault Signal Preprocessing

**Figure 5:**
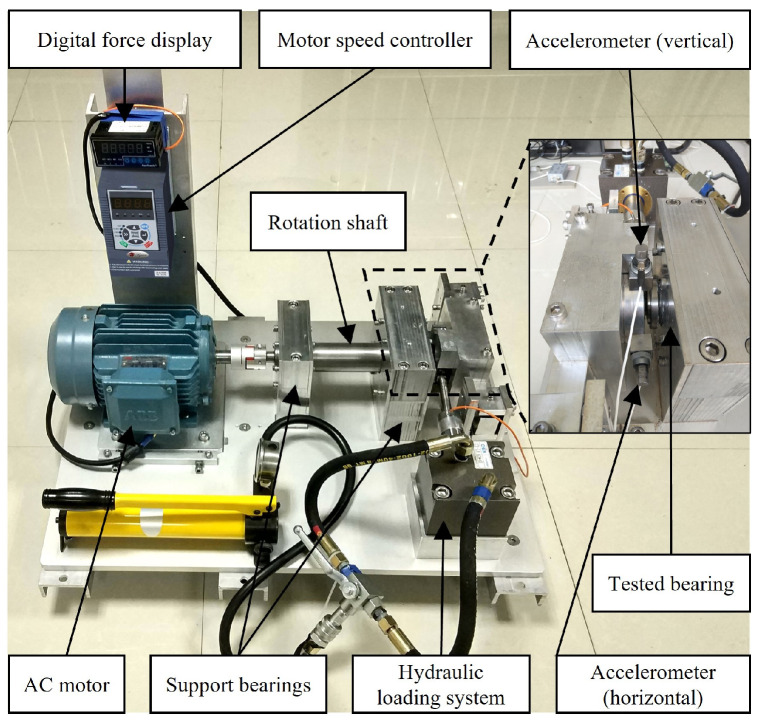
Test bench of the XJTU-SY datasets.

**Figure 6:**
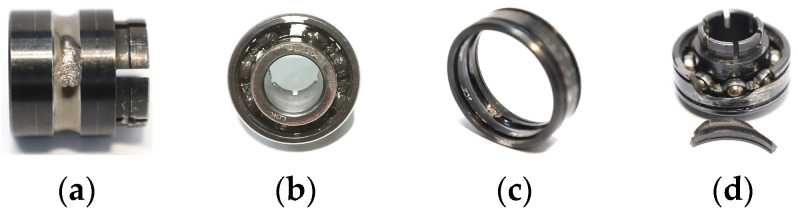
Types of degraded bearings. (**a**) Inner race wear; (**b**) cage fracture; (**c**) outer race wear; (**d**) outer race fracture.

To verify the effectiveness of the proposed method in diagnosing rolling bearing faults, experiments were conducted using the publicly available XJTU-SY rolling element bearing accelerated life test datasets [35]. The proposed method describes the entire life cycle of the bearing, from normal to various fault situations, until failure, which is suitable for research on bearing fault diagnosis and life prediction and meets experimental conditions that do not require excessive hardware configuration. Figure 5 shows the images of the test bench provided by the XJTU-SY datasets, where the model of the test bearing is LDK UER204. According to the differences in rotational speed and radial force, the dataset categorizes the test conditions into three types: a rotational speed of 2100 r/min and a radial force of 12 kN; a rotational speed of 2250 r/min and a radial force of 11 kN; and a rotational speed of 2400 r/min and a radial force of 10 kN. Under these three test conditions, the bearings experienced various failure situations, including inner race wear, cage fracture, outer race wear, and outer race fracture, as shown in Figure 6. Five bearings were tested under each operating condition, and Table 3 provides detailed information for each tested bearing in the XJTU-SY datasets.

Under the third working condition with a rotational speed of 2400 r/min and a radial force of 10 kN, the outer race of bearing 3_1 failed, and the inner race of bearing 3_4 failed. Previous studies have clearly indicated that bearings 3_1 and 3_4 exhibit different fault states throughout their entire life cycle, as shown in Table 4 [36]. In order to better demonstrate the accuracy of the proposed method in diagnosing various fault states and degrees of bearings, the full life cycle vibration data of bearings 3_1 and 3_4 were selected as test data. The data corresponding to seven groups of time were selected as the standard-state data of seven bearing states. In the full life data of bearing 3_1, four sets of data corresponding to 525, 2350, 2475, and 2538 min were selected as the standard-state data for four bearing states: normal, slight, moderate, and serious fault of the outer race. In the full life data of bearing 3_4, three sets of data corresponding to 1417, 1445, and 1479 min were selected as the standard-state data of three bearing states: slight, moderate, and serious fault of inner race. Thirty test samples were set for each of the seven states, with each sample containing 2048 data points, totaling 210 test samples.

### 4.2. Fault Signal Decomposition

**Figure 7:**
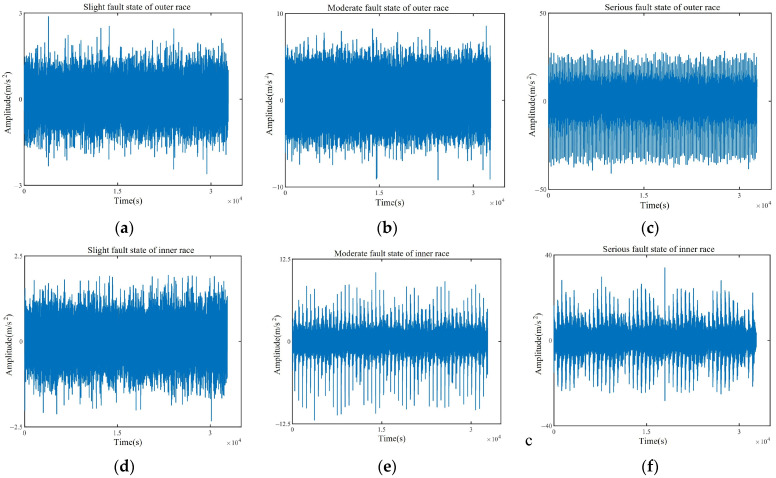
Waveforms of six fault states. (**a**) Slight fault state of outer race; (**b**) moderate fault state of outer race; (**c**) serious fault state of outer race; (**d**) slight fault state of inner race; (**e**) moderate fault state of inner race; (**f**) serious fault state of inner race.

The original signals of six fault states selected for the experiment, excluding the normal state, are shown in Figure 7. From the time-domain waveform, it can be seen that the original signals of each fault state are complex and contain a large number of impulse components. For this paper, the minimum envelope entropy was used as the objective function, and SABO was employed to determine the optimal mode number *k* and the secondary penalty factor α for the VMD decomposition of each test sample. One test sample from each state was selected as the input data for SABO. Then, we built the SABO algorithm model by setting the range of *k* to (3, 10), the range of α to (100, 2500), the variable number to be optimized *D* to 2, the population number *N* to 15, and the maximum iteration number *T* to 20. After iterations of the SABO algorithm, the optimal parameters for different fault states of rolling bearings were obtained, as shown in Table 5.

**Figure 8:**
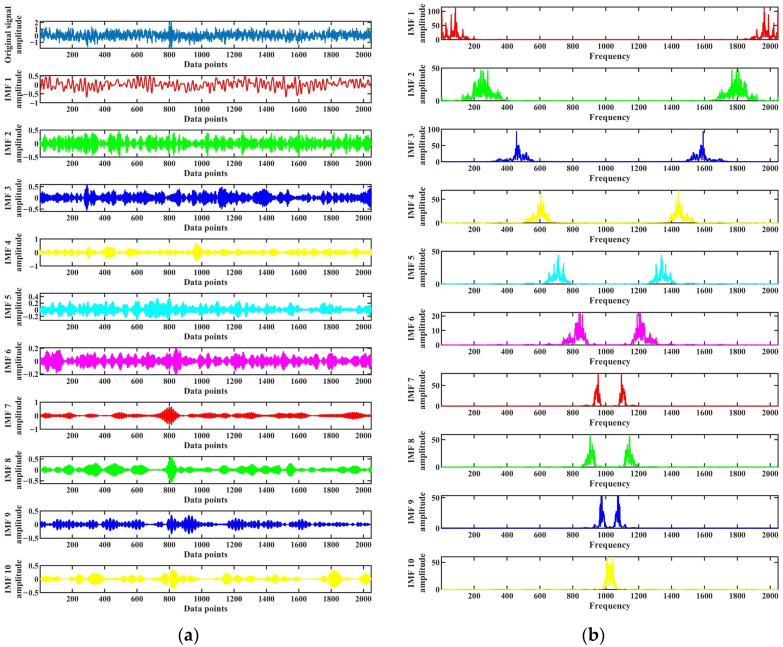
Decomposition results of the first test sample in normal state. (**a**) VMD decomposition results; (**b**) IMF component spectrums.

The optimal mode number *k* and the secondary penalty factor α optimized by the SABO algorithm were used to perform VMD decomposition on each test sample. As shown in Figure 8a, the first test sample of the normal state was optimized with the optimal parameter combination of mode number 10 and the secondary penalty factor 100. After VMD decomposition, ten IMF components were obtained, and the corresponding IMF component spectra are shown in Figure 8b. It can be seen from Figure 8a,b that the time-domain waveforms of each IMF component exhibit clear impulse characteristics, and there is no apparent modal aliasing problem in the spectrum. All frequency components have been correctly decomposed.

### 4.3. Fault Signal Reconstruction

For the multiple IMF components obtained by decomposing each test sample, the IMF component corresponding to the minimum envelope entropy was selected as the optimal IMF component. The optimal IMF components for different fault states of rolling bearings are shown in Table 6. The mean, variance, peak value, kurtosis, root mean square, peak factor, pulse factor, waveform factor, margin factor, and energy value were calculated sequentially for the optimal IMF components of each test sample. Subsequently, the feature vectors of each sample were constructed using these ten indicators. The mean of the feature vectors of all test samples in each bearing state was taken as the standard-state feature vector of the bearing state. The eigenvalues of the seven standard-state feature vectors are shown in Table 7.

### 4.4. Fault Identification and Diagnosis

**Figure 9:**
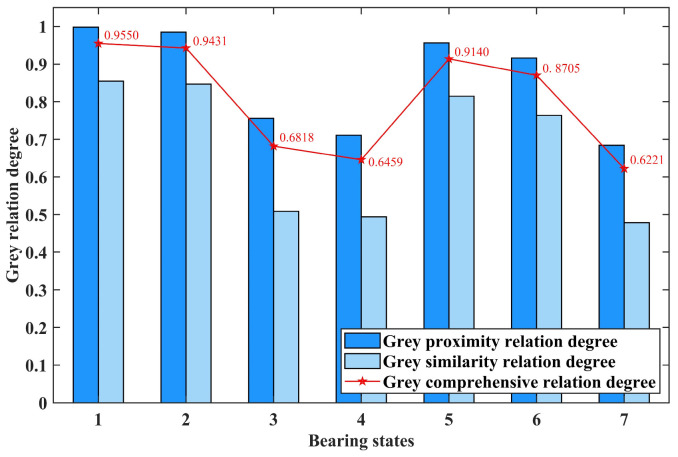
The calculation results of the first test sample in normal state.

**Figure 10:**
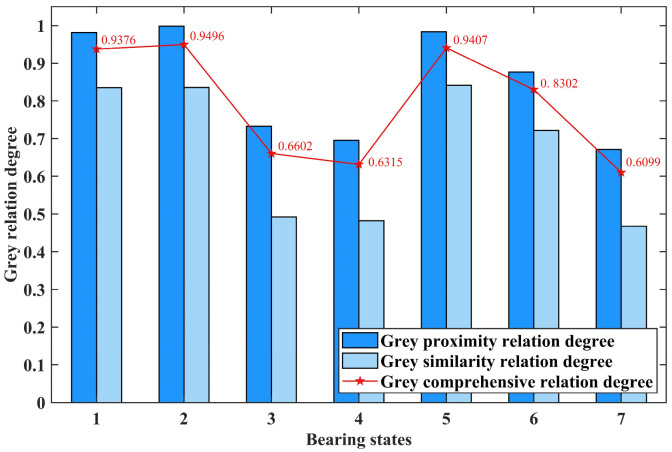
The calculation results of the first test sample in slight fault of outer race.

**Figure 11:**
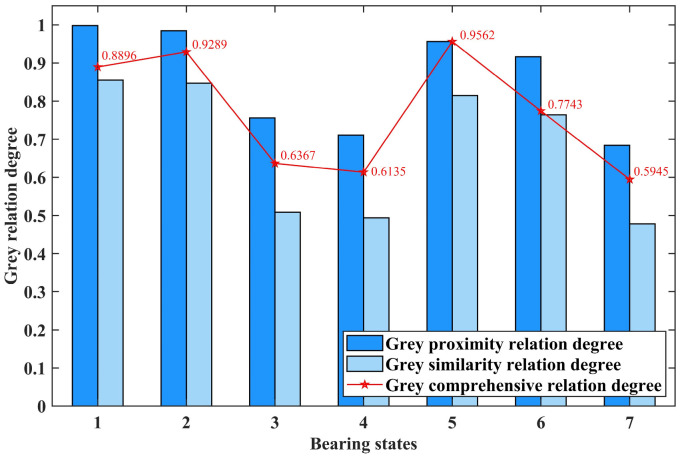
The calculation results of the first test sample in slight fault of inner race.

By calculating the grey proximity relation degree *H_ab_* and grey similarity relation degree *H*′*_a_*_′*b*′_ based on the standard distance entropy between the feature vectors of each test sample and the standard state and then weighting *H_ab_* and *H*′*_a_*_′*b*′_ with a ratio of 7:3, the grey comprehensive relation degree can be obtained. Then, compare the grey comprehensive relation degree between each test sample and each standard state. The closer the value is to 1, the closer the test sample is to a specific fault state, helping to determine the fault state of the test sample. Taking the first test sample of seven bearing states, including normal state and slight fault of outer race and inner race, as examples, the calculation results are shown in Figure 9, Figure 10 and Figure 11, respectively.

In Figure 9, Figure 10 and Figure 11, the horizontal axis represents seven different bearing states: normal state; slight, moderate, and serious fault of the outer race; and slight, moderate, and serious fault of the inner race. The vertical axis represents the calculated grey relation degree. The dark and light blue columns and the point marked by the red pentagram represent the grey proximity, similarity, and comprehensive relation degree calculated by the test sample and a specific standard bearing state feature vector, respectively.

By comparing the values calculated in Figure 9, it can be seen that the grey comprehensive relation degree of the first bearing state, which represents the normal state, is the closest to one. This indicates that the test sample is diagnosed as normal state. According to the same approach, it can be concluded that the test samples represented by Figure 10 and Figure 11 are diagnosed as slight fault state of the outer race and slight fault state of the inner race, respectively.

**Figure 12:**
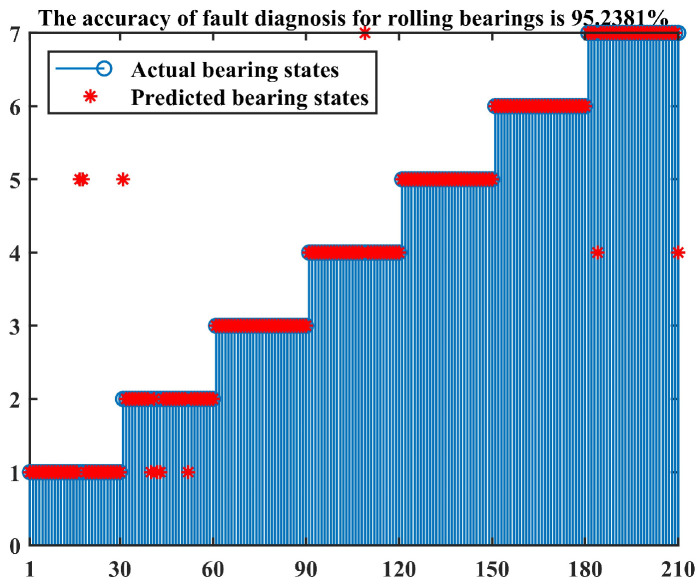
Fault diagnosis results of rolling bearings.

**Figure 13:**
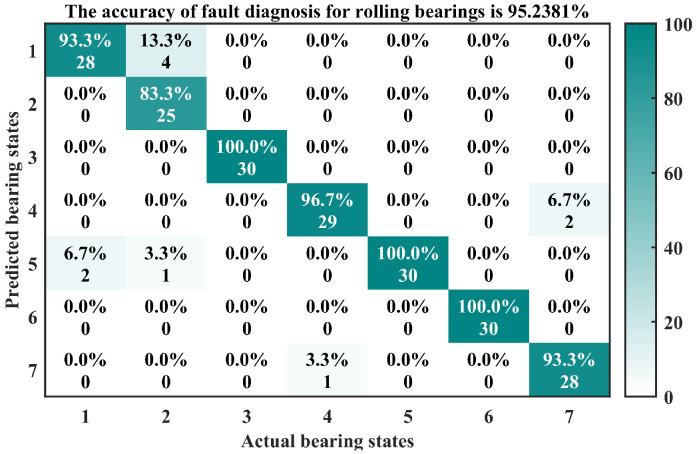
Confusion matrix results.

The confusion matrix is a common form of fully reflecting the results in the classification task. Due to its ability to clearly and intuitively display the correspondence between the predicted results and actual labels of classification models in classification tasks and the fact that it can effectively evaluate the performance of classification models, many scholars have applied it to the research of fault diagnosis [37]. For this paper, the method described above was used to calculate the grey comprehensive relation degree between 210 test samples and various standard-state feature vectors. The final diagnosis results are displayed by the confusion matrix diagrams shown in Figure 12 and Figure 13. Among them, Figure 12 is another way to show the results, which was used to compare with the confusion matrix to facilitate the understanding of the meaning of the confusion matrix diagram.

In the confusion matrix of Figure 13, the horizontal and vertical axes represent the actual and predicted bearing states, respectively. The numbers 1–7 correspond to the normal state; slight, moderate, and serious fault of outer race; and slight, moderate, and serious fault of inner race. There are a total of 49 blocks in the confusion matrix. The number above each block represents the recognition accuracy corresponding to different bearing states, while the number below represents the number of recognition samples corresponding to different bearing states.

Combined with Figure 12 and Figure 13, it can be observed that the values above the third, fifth, and sixth blocks on the diagonal of the confusion matrix are all 100%, indicating that the recognition accuracy of the third, fifth, and sixth bearing states is 100%. In the first type of bearing state, two test samples are mistakenly classified as the fifth type of bearing state (slight fault of inner race). In the second type of bearing state, four test samples are mistakenly classified as the first type of bearing state (normal), and one test sample is mistakenly classified as the fifth type of bearing state (slight fault of inner race). In the fourth type of bearing state, one test sample is mistakenly classified as the seventh type of bearing state (serious fault of inner race). In the seventh type of bearing state, two test samples are mistakenly classified as the fourth type of bearing state (serious fault of outer race). Out of 210 test samples, a total of 200 samples were correctly identified, and the method proposed in this paper ultimately achieved a diagnostic accuracy of 95.24%.

### 4.5. Accuracy Comparison of Different Algorithms

The fault diagnosis method for rolling bearings based on grey relation degree proposed in this paper is based on efficient mathematical operation and does not need complex hardware acceleration support. The calculation process of fault signal preprocessing, decomposition, reconstruction, fault identification, and diagnosis were realized by using the platform named MATLAB R2022a. To validate the superiority of the method proposed in this paper, 210 sets of test sample data were input into other algorithm models for calculation, and accuracy results were obtained for a comparative analysis. The first diagnostic method combines GWO-VMD with grey relation degree. The GWO algorithm was used to replace the SABO algorithm to optimize the mode number and the secondary penalty factor of VMD decomposition. The vibration signals of rolling bearings were decomposed using VMD based on the optimized parameter results. Then, with the minimum envelope entropy as the objective function, the feature vectors of the optimal IMF component of all test samples were extracted (using the same ten eigenvalues as the method used in this paper) and input into the grey relation degree model for fault diagnosis.

The second diagnostic method is GWO-VMD-KELM, which inputs the feature vectors of all test samples extracted by the first method into the KELM model for fault diagnosis. In addition, the feature vectors of all test samples extracted by the SABO-VMD method were input into the KELM model to form the third diagnostic method, SABO-VMD-KELM. Finally, we replaced the KELM method with SVM to form the fourth diagnostic method, GWO-VMD-SVM, and the fifth diagnostic method, SABO-VMD-SWM.

**Figure 14:**
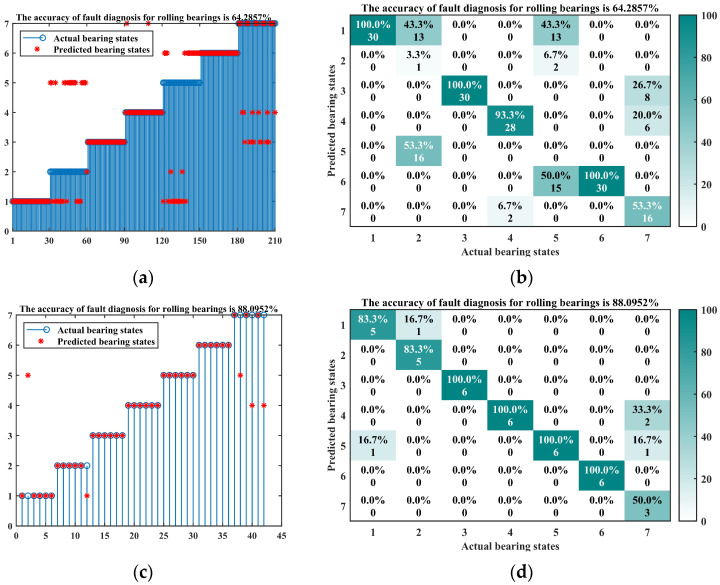

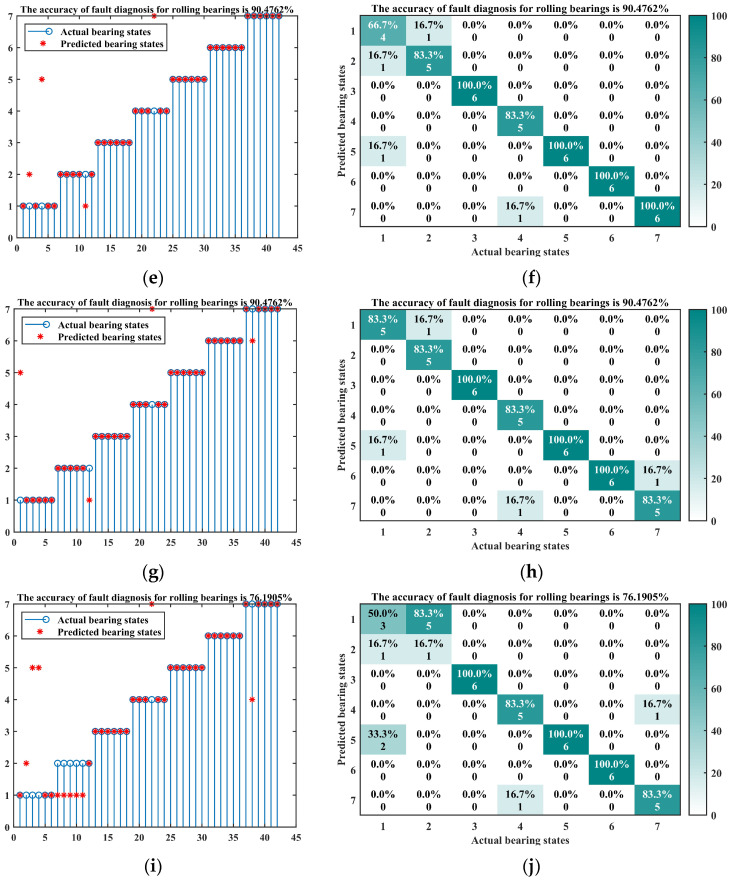
The diagnostic results and confusion matrix results of the three diagnostic methods. (**a**) Diagnosis result of GWO-VMD combined with grey relation degree; (**b**) confusion matrix of GWO-VMD combined with grey relation degree; (**c**) diagnosis result of GWO-VMD-KELM; (**d**) confusion matrix of GWO-VMD-KELM; (**e**) diagnosis result of SABO-VMD-KELM; (**f**) confusion matrix of SABO-VMD-KELM; (**g**) diagnosis result of GWO-VMD-SVM; (**h**) confusion matrix of GWO-VMD-SVM; (**i**) diagnosis result of SABO-VMD-SVM; (**j**) confusion matrix of SABO-VMD-SVM.

In the following four diagnostic methods, there were thirty samples for each bearing state, of which the top 80% were used as training sets, while the remaining 20% were used as test sets. The diagnostic results and confusion matrix results of these five diagnostic methods are shown in Figure 14. A summary of the five calculation methods and the fault diagnosis method for rolling bearings based on grey relation degree proposed in this paper is shown in Table 8.

Considering Figure 14 and Table 8, it can be seen that the method of using GWO algorithm to optimize VMD decomposition and using grey relation degree for fault diagnosis has 75 incorrectly identified samples, corresponding to an accuracy of 64.29%. Figure 14 and Table 8 also reflect that the SABO algorithm used in this paper has more advantages in optimizing the parameters of VMD compared to the GWO algorithm. The methods of GWO-VMD-KELM, SABO-VMD-KELM, GWO-VMD-SVM, and SABO-VMD-SVM, which use artificial intelligence algorithms for fault diagnosis, have accuracy rates of 88.10%, 90.48%, 90.48%, and 76.49%, respectively. Although both are higher than the method of GWO-VMD combined with the grey relation method, there are still many incorrect samples, indicating that the use of artificial intelligence algorithms cannot significantly improve fault diagnosis accuracy. It is necessary to carry out a extensive sample training and integrate some methods to optimize the structural parameters of artificial intelligence algorithms. Through the comparison of different algorithms, the fault diagnosis method for rolling bearings based on grey relation degree proposed in this paper was found to have the highest diagnostic accuracy and the most effective identification ability. It does not require a large number of training samples, and its relatively low hardware requirements provide more options for practical applications and reduce cost risks.

## 5. Conclusions

Aiming at the difficult problem of the extraction of fault characteristics and low accuracy of fault diagnosis throughout the full life cycle of rolling bearings, a fault diagnosis method for rolling bearings based on grey relational degree is proposed in this paper. The correctness and effectiveness of the proposed method were verified by using the publicly available XJTU-SY rolling element bearing accelerated life test datasets. Our main conclusions are as follows:

(1) The method proposed in this paper abandons the original method of manually setting VMD parameters. The minimum envelope entropy was taken as the objective function, and the SABO algorithm was used to optimize the two important parameters of VMD: mode number and the secondary penalty factor. The optimization results were used to decompose the vibration signal by VMD, avoiding the problems of under-decomposition and over-decomposition, caused by improper parameter settings. Moreover, it is more convenient for extracting fault features and optimizing the parameters of rolling bearings.
(2) Traditional grey relation models cannot accurately reflect the similarity and proximity between two curves, resulting in significant errors. The grey proximity and similarity relation model based on standard distance entropy were weighted to calculate the grey comprehensive relation degree between the feature vector of vibration signal and the standard state. By comparing the values of the calculation results, the diagnosis of different fault states and degrees of rolling bearings were realized.
(3) By using the XJTU-SY rolling element bearing accelerated life test datasets for testing, a fault diagnosis accuracy of 95.24% was achieved. By comparing the proposed method with various other feature extraction and fault diagnosis methods, it can be seen that the proposed method does not need to consider the acceleration support of hardware devices or carry out extensive sample training like artificial intelligence algorithms, nor does it design a model with a complex network structure. It can achieve high fault recognition accuracy, has better feasibility, and has a higher practical engineering application value.

Rolling bearings are required to ensure high-precision continuous operation in various complex and harsh environments. In this study, the accuracy of fault diagnosis for rolling bearings under operating conditions with a rotational speed of 2400 r/min and a radial force of 10 kN was found to be very high. However, the recognition and diagnosis of fault states under various complex working conditions, such as variable speeds, are still unknown, and further research and exploration are needed.

## Figures and Tables

**Table 1:** Test functions in CEC2005.

Sequence Number	Test Function	Search Space
F1	$F_{1}(x)=\sum_{i=1}^{n}x_{i}^{2}$	[−100, 100]
F2	$F_{2}(x)=\sum_{i=1}^{n}\left|x_{i}\right|+\prod_{i=1}^{n}\left|x_{i}\right|$	[−10, 10]
F9	$F_{9}(x)=\sum_{i=1}^{n}\left[x_{i}^{2}-10\cos(2\pi x_{i})+10\right]$	[−5.12, 5.12]
F10	$F_{10}(x)=-20\exp\left(-0.2\sqrt{\frac{1}{n}\sum_{j=1}^{n}x_{j}^{2}}\right)-\exp\left(\frac{1}{n}cos\left(2\pi x_{j}\right)\right)+20+e$	[−32, 32]

**Table 2:** Equations of characteristic parameters.

Feature Parameter	Equation	Feature Parameter	Equation
Mean	$\bar{X}=\frac{1}{N}\sum_{i=1}^{N}x_{i}$	Peak factor	$I_{p}=\frac{X_{p}}{X_{rms}}$
Variance	$X^{2}=\frac{1}{N}\sum_{i=1}^{N}{\left(x_{i}-\bar{X}\right)}^{2}$	Pulse factor	$I_{i}=\frac{X_{p}}{\left|\bar{X}\right|}$
Peak value	$X_{p}=X_{max}-X_{min}$	Waveform factor	$I_{\omega }=\frac{X_{rms}}{\left|\bar{X}\right|}$
Kurtosis	$X_{kur}=\frac{\sum_{i=1}^{N}{\left(x_{i}-\bar{X}\right)}^{4}}{\left(N-1\right)X_{\sigma }^{4}}$	Margin factor	$I_{m}=\frac{X_{p}}{{\left[\frac{1}{N}\sum_{i=1}^{N}\sqrt{\left|x_{i}\right|}\right]}^{2}}$
Root mean square	$X_{rms}=\sqrt{\frac{1}{N}\sum_{i=1}^{N}x_{i}^{2}}$	Energy value	$E_{i}={\int_{-\infty }^{+\infty }\left|x_{i}\left(t\right)\right|}^{2}dt$

**Table 3:** Detailed information for each tested bearing in the XJTU-SY datasets.

Operating Conditions	Tested Bearing	Actual Life	Failure Location
Condition 1: a rotational speed of 2100 r/min and a radial force of 12 kN	Bearing1_1	123 min	Outer race
	Bearing1_2	161 min	Outer race
	Bearing1_3	158 min	Outer race
	Bearing1_4	122 min	Cage
	Bearing1_5	52 min	Inner race, outer race
Condition 2: a rotational speed of 2250 r/min and a radial force of 11 kN	Bearing2_1	491 min	Inner race
	Bearing2_2	161 min	Outer race
	Bearing2_3	533 min	Cage
	Bearing2_4	42 min	Outer race
	Bearing2_5	339 min	Outer race
Condition 3: a rotational speed of 2400 r/min and a radial force of 10 kN	Bearing3_1	2538 min	Outer race
	Bearing3_2	2496 min	Inner race, outer race, rolling element, cage
	Bearing3_3	371 min	Inner race
	Bearing3_4	1515 min	Inner race
	Bearing3_5	114 min	Outer race

**Table 4:** The bearing status corresponding to different times during operation.

Tested Bearing	Failure Location	Operating Time	Bearing State
Bearing3_1	Outer race	525 min	Normal
		2350 min	Slight fault of outer race
		2475 min	Moderate fault of outer race
		2538 min	Serious fault of outer race
Bearing3_4	Inner race	1417 min	Slight fault of inner race
		1445 min	Moderate fault of inner race
		1479 min	Serious fault of inner race

**Table 5:** Optimal VMD parameters for different bearing states.

Bearing State	Normal	Fault State of Outer Race			Fault State of Inner Race		
		Slight	Moderate	Serious	Slight	Moderate	Serious
Mode number	10	10	10	4	4	10	10
Secondary penalty factor	100	117	2500	2084	2500	2500	1585

**Table 6:** Optimal IMF components for different fault states of rolling bearings.

Bearing State	Normal	Fault State of Outer Race			Fault State of Inner Race		
		Slight	Moderate	Serious	Slight	Moderate	Serious
Optimal IMF components	IMF8	IMF8	IMF10	IMF2	IMF1	IMF2	IMF2

**Table 7:** Feature vectors of seven standard states.

	State	Normal	Fault State of Outer Race			Fault State of Inner Race		
Eigenvalues			Slight	Moderate	Serious	Slight	Moderate	Serious
Mean		4.49 × 10^−5^	7.28 × 10^−7^	−3.70 × 10^−5^	5.07 × 10^−4^	−3.27 × 10^−3^	−1.22 × 10^−3^	5.80 × 10^−3^
Variance		0.011	0.012	0.013	1.225	0.293	0.096	0.086
Peak value		0.717	0.731	0.758	9.518	3.705	1.935	1.942
Kurtosis		3.356	3.312	3.376	6.063	3.607	3.415	3.487
Root mean square		0.104	0.107	0.114	1.093	0.541	0.307	0.285
Peak factor		6.857	6.781	6.704	8.774	6.841	6.365	6.781
Pulse factor		8.729	8.612	8.590	12.896	8.917	8.257	8.810
Waveform factor		1.267	1.269	1.277	1.454	1.300	1.295	1.296
Margin factor		10.373	10.222	10.272	16.654	10.782	9.968	10.643
Energy value		24.290	45.314	338.429	2508.161	600.911	195.779	175.242

**Table 8:** Fault diagnosis accuracy of different algorithms.

Feature Extraction Method	Fault Diagnosis Method	Accuracy
GWO-VMD	Grey relation degree	64.29%
GWO-VMD	KELM	88.10%
SABO-VMD	KELM	90.48%
GWO-VMD	SVM	90.48%
SABO-VMD	SVM	76.19%
SABO-VMD	Grey relation degree	95.24%

## Data Availability

The data used in this study are openly available in XJTU-SY datasets at http://biaowang.tech/xjtu-sy-bearing-datasets (accessed on 7 September 2023).

## References

[1] Roy S.S., Dey S., Chatterjee S. (2020). Autocorrelation aided random forest classifier-based bearing fault detection framework. IEEE Sens. J..

[2] Rostaghi M., Khatibi M.M., Ashory M.R., Azami H. (2023). Refined composite multiscale fuzzy dispersion entropy and its applications to bearing fault diagnosis. Entropy.

[3] Lin F., Chai J., Cao Y.F., Yang D.H., Zang L.G. (2022). Prediction and analysis of bending fatigue life of hub bearing considering oil film lubrication. Lubr. Eng..

[4] Choudhary A., Mian T., Fatima S., Panigrahi B.K. (2023). Fault diagnosis of electric two-wheeler under pragmatic operating conditions using wavelet synchrosqueezing transform and CNN. IEEE Sens. J..

[5] Liang P., Deng C., Wu J. (2020). Intelligent fault diagnosis via semisupervised generative adversarial nets and wavelet transform. IEEE Trans. Instrum. Meas..

[6] Alfarizi M.G., Tajiani B., Vatn J., Yin S. (2023). Optimized random forest model for remaining useful life prediction of experimental bearings. IEEE Trans. Ind. Inform..

[7] Maurya S., Singh V., Verma N.K. (2020). Condition monitoring of machines using fused features from EMD-based local energy with DNN. IEEE Sens. J..

[8] Habbouche H., Amirat Y., Benkedjouh T., Benbouzid M. (2022). Bearing fault event-triggered diagnosis using a variational mode decomposition-based machine learning approach. IEEE Trans. Energy Convers..

[9] Heydari A., Garcia D.A., Fekih A., Keynia F., Tjernberg L.B., Santoli L.D. (2021). A hybrid intelligent model for the condition monitoring and diagnostics of wind turbines gearbox. IEEE Access.

[10] Wang X.H., Sui G.Z., Xiang J.W., Wang G.B., Huo Z.Q., Huang Z. (2020). Multi-domain extreme learning machine for bearing failure detection based on variational modal decomposition and approximate cyclic correntropy. IEEE Access.

[11] Lv M.Z., Liu S.X., Chen C.Z. (2022). A new feature extraction technique for early degeneration detection of rolling bearings. IEEE Access.

[12] Yuan H.Y., Wang X.Y., Sun X. (2017). Compressive sensing-based feature extraction for bearing fault diagnosis using a heuristic neural network. Meas. Sci. Technol..

[13] Medina R., Macancela J.C., Lucero P. (2022). Gear and bearing fault classification under different load and speed by using Poincaré plot features and SVM. J. Intell. Manuf..

[14] Jia M.S., Qi Z.Y., Xue D.Q. (2023). The fault prediction of bearing based on GASA-BP-BiLSTM. Modul. Mach. Tool Autom. Manuf. Tech..

[15] Cao J., Zhang Y.L., Wang J.H., Yu P. (2022). Fault diagnosis of rolling bearing based on VMD and SVPSO-BP. Acta Energiae Solaris Sin..

[16] Liu J., Li C.J., Zhao X., Tan Y.T. (2023). Rolling bearing fault diagnosis based on multi-feature fusion and GSA-SVM. Chin. J. Sens. Actuators.

[17] Abualigah L., Diabat A., Mirjalili S., Elaziz M.A., Gandomi A.H. (2021). The arithmetic optimization algorithm. Comput. Methods Appl. Mech. Eng..

[18] Chen J., Yang H.J., Ji L., Xu T.L., Huang Z., Li X.Y. (2023). Method of bearing fault diagnosis based on SVM optimized by AOA algorithm. Electron. Meas. Technol..

[19] Zhu L.W., Tian X., Li X.H. (2023). Fault identification modal and its application based on ISAM-Drsnet. J. Mech. Electr. Eng..

[20] Liu S.F., Tao Y., Xie N.M., Tao L.Y., Hu M.L. (2022). Advance in grey system theory and applications in science and engineering. Grey Syst..

[21] Wei B.L., Xie N.M. (2019). Unified representation and properties of generalized grey relational analysis models. Syst. Eng.-Theory Pract..

[22] Darvishi D., Liu S.F., Jeffrey Y.L.F. (2021). Grey linear programming: A survey on solving approaches and applications. Grey Syst..

[23] Lian B.X., Yan B., Deng Z.M., Ke S. (2023). Early fault diagnosis for rolling bearings based on DDAE-GRA. Bearing.

[24] Peng Z.H., Song B. (2010). Research on fault diagnosis method for transformer based on fuzzy genetic algorithm and artificial neural network. Kybernetes.

[25] Lin F., Tang J., Zhao Y.Q., Li J.L., Zang L.G., Chen Y.K. (2020). Load distribution and bending fatigue life analysis of hub bearings based on modified L-P model. China Mech. Eng..

[26] Lu L., Wang W., Kong D., Zhu J., Chen D. (2023). Fault diagnosis of rotating machinery using kernel neighborhood preserving embedding and a modified sparse bayesian classification model. Entropy.

[27] Dragomiretskiy K., Zosso D. (2014). Variational mode decomposition. IEEE Trans. Signal Process..

[28] Pavithra R., Ramachandran P. (2023). Deep convolution neural network for machine health monitoring using spectrograms of vibration signal and its EMD-intrinsic mode functions. J. Intell. Fuzzy Syst..

[29] Pavel T., Mohammad D. (2023). Subtraction-average-based optimizer: A New swarm-inspired metaheuristic algorithm for solving optimization problems. Biomimetics.

[30] Souaidia C., Thelaidia T., Chenikher S. (2023). Independent vector analysis based on binary grey wolf feature selection and extreme learning machine for bearing fault diagnosis. J. Supercomput..

[31] Nayana B.R., Geethanjali P. (2020). Improved identification of various conditions of induction motor bearing faults. IEEE Trans. Instrum. Meas..

[32] Brusamarello B., Da Silva J.C.C., De Morais Sousa K., Guarneri G.A. (2023). Bearing fault detection in three-phase induction motors using support vector machine and fiber bragg grating. IEEE Sens. J..

[33] Liu X.M., Xie N.M. (2022). Grey-based approach for estimating software reliability under nonhomogeneous Poisson process. J. Syst. Eng. Electron..

[34] Lu N.N., Liu S.F., Du J.L. (2023). Grey relational analysis model with cross-sequences and its application in evaluating air quality index. Expert Syst. Appl..

[35] Wang B., Lei Y.G., Li N.P., Li N.B. (2018). A hybrid prognostics approach for estimating remaining useful life of rolling element bearings. IEEE Trans. Reliab..

[36] Yan X.A., Jia M.P. (2021). Intelligent fault diagnosis of rolling element bearing using hierarchical multiscale dispersion entropy. Trans. Chin. Soc. Agric. Eng..

[37] Kevin R., Michael N., Martin H. (2023). Hierarchical confusion matrix for classification performance evaluation. arXiv.

